# IL-35-Producing Regulatory B Cells Are Positively Correlated with iTr35 Cells in Healthy Pregnancy with Altered Associations Following Pregnancy Loss

**DOI:** 10.3390/ijms27209207

**Published:** 2026-10-16

**Authors:** Anna Slawek, Paulina Kubik, Daria Lorek, Anna Ewa Kedzierska, Mateusz Spetany, Urszula Studzinska-Kalota, Anna Chelmonska-Soyta

**Affiliations:** 1 Hirszfeld Institute of Immunology and Experimental Therapy, Polish Academy of Sciences, 53-114 Wrocław, Poland; paulina.kubik@hirszfeld.pl (P.K.); anna.kedzierska@hirszfeld.pl (A.E.K.); soyta@hirszfeld.pl (A.C.-S.); 2 Department of Immunology, Pathophysiology and Veterinary Preventive Medicine, Faculty of Veterinary Medicine, Wrocław University of Environmental and Life Sciences, 50-375 Wrocław, Poland; daria.lorek@upwr.edu.pl; 3 Gynaecology and Obstetrics Department, Antoni Falkiewicz Specialist Hospital, 52-114 Wrocław, Poland; mspetany@gmail.com; 4 Healthcare Facility “Healthy Woman”, 54-034 Wrocław, Poland; studzinska@post.pl

**Keywords:** IL-35, iTr35 cells, Bregs, Tregs, B10 cells, PD-L1, PD-L2, RPL, pregnancy

## Abstract

Immunological dysregulation may be involved in pregnancy loss. Regulatory T (Treg) and B (Breg) cells, together with interleukin-35 (IL-35), contribute to maternal-fetal tolerance. This study investigated peripheral regulatory cell populations in healthy pregnancy and recurrent spontaneous pregnancy loss (RPL), focusing on the potential relationships between regulatory B and T cells within a coordinated immunoregulatory network associated with immune tolerance during pregnancy. Peripheral blood samples were collected from healthy pregnant women and women following spontaneous miscarriage. Frequencies of Breg subsets, including B10 cells, immature B cells, and plasmablasts, as well as the proportions of cells expressing IL-35, IL-10, PD-L1, and PD-L2, were analyzed. Tregs and the regulatory T-cell subsets iTr35, Tr1, and Th3 were also examined for the expression of IL-35, IL-10, TGF-β, and PD-1. In addition, correlations between Treg and Breg populations were assessed. Miscarriage was associated with significantly reduced frequencies of IL-35^+^ immature B cells, IL-35^+^ B10-like cells, IL-35^+^ plasmablasts, IL-35^+^ Tregs, and iTr35 cells. A negative correlation was observed between PD-L1-expressing B10-like cells and the frequencies of Treg and Th3 cells. Notably, this study is the first to demonstrate a positive correlation between IL-35-producing Bregs and iTr35 cells in healthy pregnancy. These findings suggest that the observed relationships between peripheral regulatory B- and T-cell populations and IL-35-associated regulatory subsets may be relevant to immune regulation during pregnancy. The iTr35/IL-35^+^ Breg ratios showed potential discriminatory value between healthy pregnancy and pregnancy loss groups and may warrant further investigation as candidate markers in prospective studies.

## 1. Introduction

According to the Practice Committee of the American Society for Reproductive Medicine (ASRM) and the European Society of Human Reproduction and Embryology (ESHRE), miscarriage affects approximately 3–5% of couples trying to conceive. Most miscarriages are sporadic and caused by genetic factors. Recurrent pregnancy loss (RPL) is defined as two or more failed clinical pregnancies. Approximately 50% of these cases are idiopathic without a clearly defined etiology [1,2]. Several etiological factors for recurrent miscarriages have been described, including genetic defects, anatomical abnormalities, endocrine disorders, infections, thrombophilia, and environmental exposure [3]. Immunological dysregulation [1,4] can be considered a potential etiological factor of RPL.

However, regardless of the factors causing miscarriage, its immunological effect is the disruption or abolition of tolerance in favor of pro-inflammatory and regenerative reactions [4,5]. Understanding the complex mechanisms of fetomaternal tolerance is critical for preventing fetal immune rejection and abortion. During pregnancy, the mother’s immune system must adapt to foreign fetal antigens and simultaneously defend her against infections. This compromise requires the cooperation of numerous immune cells, their interactions and interdependencies, and an appropriate profile of secreted cytokines. Successful pregnancies also depend on timely and efficient transitions from the pro- to anti-inflammatory stages. Although the changes in the number and phenotype of cytokine-producing lymphocytes during normal pregnancy are observed mainly in the maternal-fetal interface locally in the uterus, peripheral adaptations are also visible and correlate with the type and strength of paternal antigen expression [6,7,8,9]. Moreover, in pathological pregnancies, their repertoire is altered, leading to cellular and cytokine imbalances [10].

Immune regulatory cells are crucial for the induction of maternal tolerance and pregnancy success [11]. Regulatory T cells (Tregs) are the most well-characterized regulatory cells. While Foxp3^+^ Tregs represent the most well-characterized population, they are supported by specialized inducible T cell subsets, including inducible regulatory T cells producing interleukin-35 (IL-35; iTr35), Tr1 cells producing high levels of interleukin-10 (IL-10), and Th3 cells producing transforming growth factor-β (TGF-β) [12]. A healthy pregnancy is marked by the expansion of Tregs both within the uterine environment and in the maternal bloodstream [13]. Their immunosuppressive function is exerted, among other mechanisms, through direct cell–cell interactions with the target cell, and the release of anti-inflammatory cytokines, such as IL-35, IL-10 and TGF-β [14]. Unexplained infertility and miscarriage are associated with numerical and functional Treg deficiencies [15,16,17]. Adoptive transfer of Tregs from normal pregnant mice to abortion-prone mice prevented fetal rejection [18]. In the human decidua, regulatory B cells (Bregs) are found in close proximity to Tregs. Bregs and Tregs formed clusters, suggesting functional interactions between these cell populations [19].

In contrast to Tregs, the role of Bregs in supporting healthy pregnancy remains poorly understood. Bregs are a subset of B cells that do not constitute a uniform population because they may exhibit different phenotypes. To date, no single marker has been universally accepted for identifying all Bregs [20]. Bregs have immunosuppressive functions and modulate the immune system to prevent excessive inflammation by regulating T cell responses [21] through cell-to-cell contact involving inhibitory molecules, such as programmed cell death ligand-1 (PD-L1), programmed cell death ligand-2 (PD-L2) [22,23], and TIGIT [24], as well as by producing anti-inflammatory cytokines, including IL-10 and IL-35 [25]. Inhibitory receptors modulate immune cell activation, limit immune responses, and preserve self-tolerance [26]. Given these regulatory functions, alterations in Breg populations may have significant consequences for pregnancy. Indeed, in women with RPL, both the proportion of CD19^+^ IL-10^+^ B cells and systemic IL-10 concentration were reduced. Additionally, these women exhibited lower IL-10 and PD-L1 mRNA expression levels in in vitro–stimulated B cells [27]. Importantly, another study demonstrated that PD-L1 may play an important role in maintaining pregnancy, as it is expressed in immune cell populations in both the periphery and decidua [28], and its expression is upregulated in syncytiotrophoblasts and intermediate trophoblasts [29]. The role of the second ligand of the PD-1 receptor, PD-L2, in pregnancy is less well characterized [30]. Therefore, Bregs and their inhibitory receptor expression may be important factors contributing to the development of maternal-fetal immune tolerance and maintenance of pregnancy. In addition, IL-10^+^ Bregs can produce the immunosuppressive cytokine IL-35 [31,32], which is primarily secreted by Tregs and iTr35 cells [33,34]. IL-35 not only directly suppresses T cell effector functions and proliferation [35], but also promotes regulatory responses [36,37]. Furthermore, IL-35 produced by Tregs inhibits the capacity of CD4^+^ T cells to differentiate into Th17 effector cells [38]. Previous studies have demonstrated an increased frequency of B10 cells during normal pregnancy in both humans and murine models, whereas this population was reduced in abortion-prone pregnancies, suggesting a potential role for B10 cells in the maintenance of maternal-fetal immune tolerance [39]. Consistent with these findings, the percentages of CD24^hi^CD38^hi^ Bregs and CD24^hi^CD27^+^ Bregs, which have been reported to have IL-10-producing potential, were shown to be increased in the human decidua compared with the mid-luteal phase endometrium, further supporting the presence of regulatory B-cell populations at the maternal–fetal interface [40]. Importantly, Liu et al. demonstrated that IL-35 promotes the generation of IL-35-producing Breg cells and enhances the expansion of B10 cells, providing evidence for a functional link between IL-35 and Breg-mediated immune regulation during pregnancy. Moreover, in an abortion-prone mouse model, we confirmed the tolerogenic potential of IL-35 in pregnancy, demonstrating that a single administration of IL-35 shortly after mating increased the frequency of B10 cells (CD19^+^CD5^+^CD1d^hi^), IL-35-producing B10 cells, Tregs, and IL-35-producing Tregs in the periphery, as well as CD19^+^IL-35^+^ B cells in the decidua [41]. The placental expression of IL-35 and its components p35 and Ebi3 differs between mice abortion-prone (AP; CBA/J♀ × DBA/2J♂) and normal pregnancy (NP; CBA/J♀ × Balb/c♂) groups. The lower IL-35 expression detected in AP females suggests that IL-35 is involved in regulating pregnancy at the maternal-fetal interface [42]. Furthermore, in our previous study, using the same murine model, we demonstrated a decreased frequency of CD19^+^IL-35^+^ cells in the uterus on day 3 and in the decidua on day 14 of pregnancy [32]. This work highlights both the immunosuppressive mechanisms of regulatory cell action, exerted through direct cell-to-cell interactions, and the secretion of anti-inflammatory cytokines.

Therefore, the aim of this study was to provide a detailed phenotypic characterization of peripheral blood regulatory cells, with particular emphasis on IL-35 subunits and the inhibitory molecule PD-L1 in these cells in normal pregnancy and during spontaneous abortion. This work constitutes a new insight into maternal immune adaptation as a consequence of miscarriage in the context of changes in frequencies of lymphocytes with regulatory potential. This work also highlights the potential importance of the coordinated regulatory network involving Tregs and Bregs in successful pregnancy. Non-invasive peripheral blood examination may be useful additional source of information on immune adaptive remodeling in women after miscarriage in compare with women in normal pregnancy.

## 2. Results

### 2.1. Increased B10-like Cell Proportions and Decreased the Plasmablast Frequencies in Women with RPL

**Figure 1:**
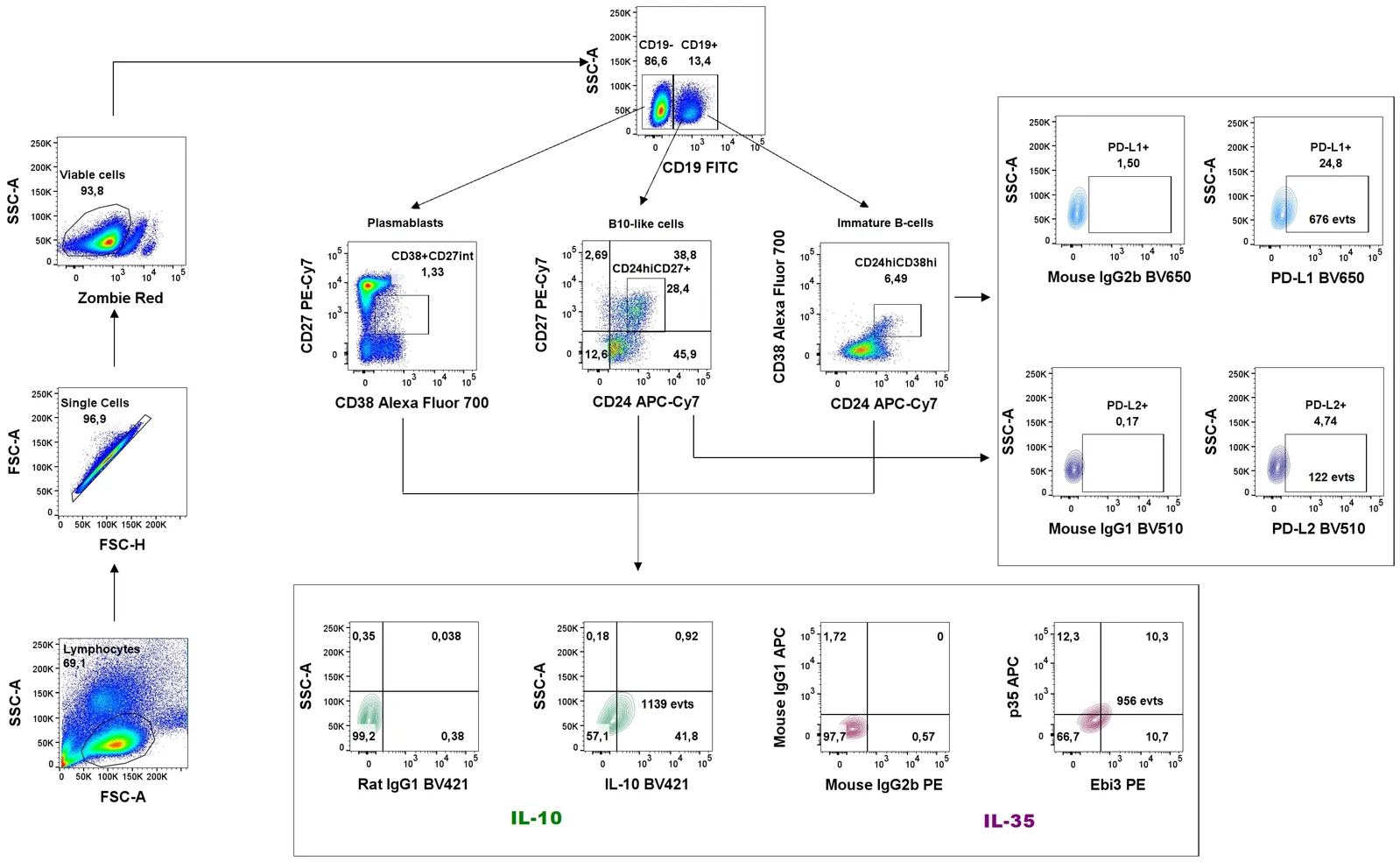
Representative dot-plots showing the gating strategy of regulatory B cells: B10-like cells (CD19^+^CD24^high^CD27^+^), immature B-cells (CD19^+^CD24^high^CD38^high^), and plasmablasts (CD19^−^CD27^int^CD38^+^) from representative pregnancy loss samples, expressing PD-L1 and PD-L2, as well as producing IL-35 and IL-10. The same gating strategy was applied to all three B-cell populations in all study groups. IL-35-producing cells were defined based on intracellular co-expression of its two subunits, Ebi3 and p35. For cytokine analysis and PD-L1 and PD-L2 molecules, we used appropriate isotype controls in the same concentration as specific antibodies. For other surface antigens, FMO controls were used. The numbers of events in the terminal gates are indicated. Representative flow-cytometry plots illustrating B10-like cell subpopulations’ resolution in the study groups are presented in Supplementary Data (Figure S1).

**Figure 2:**
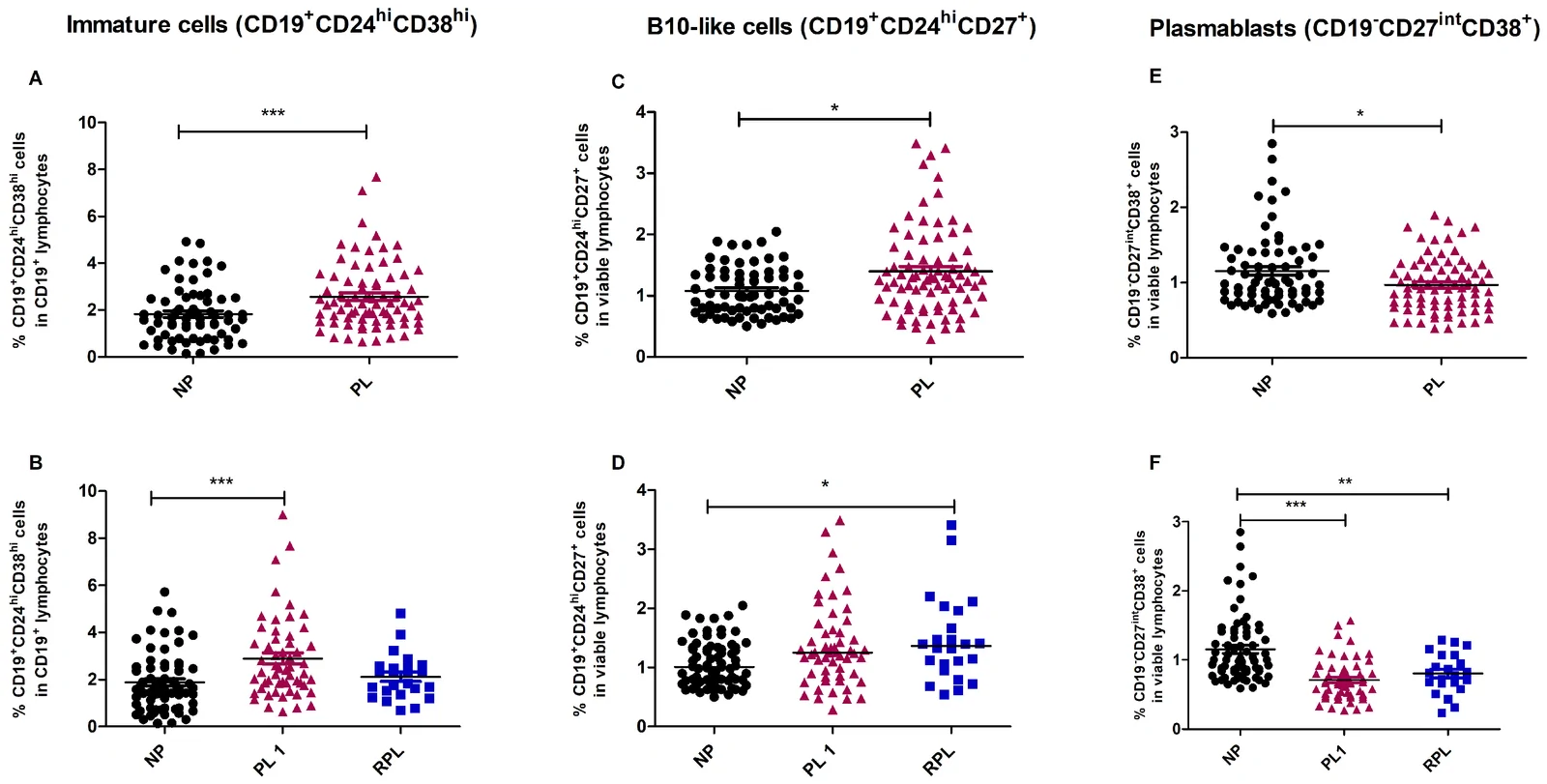
The percentage of immature cells within CD19^+^ lymphocytes (**A**,**B**), B10-like cells (**C**,**D**) and plasmablasts (**E**,**F**) within viable lymphocytes in peripheral venous blood from healthy women (NP—normal pregnancy, *n* = 68) and women after miscarriages (PL—pregnancy loss (total), *n* = 79; PL1—group of women who experienced one miscarriage, *n* = 56; RPL—group of women affected by 2–4 miscarriages, *n* = 23). Data are expressed as mean ± standard error of the mean (SEM). Normality was assessed using the Shapiro–Wilk normality or Kolmogorov–Smirnov tests. * *p* < 0.05, ** *p* < 0.01, *** *p* < 0.001 as analyzed by Student’s *t*-test (parametric) or Mann–Whitney U test (non-parametric) to compare two groups or one-way analysis of variance with Tukey’s multiple comparison test (parametric) or Kruskal–Wallis test with Dunn’s multiple comparison test (non-parametric) to compare three groups.

Based on the gating strategy depicted in Figure 1, the following Breg cell phenotypes were identified: B10-like cells (CD19^+^CD24^high^CD27^+^), immature B cells (CD19^+^CD24^high^CD38^high^), and plasmablasts (CD19^−^CD27^int^CD38^+^) in healthy pregnant women (NP, *n* = 68) and women with pregnancy loss in the first trimester of pregnancy (PL, *n* = 79), including women who experienced a single miscarriage (PL1, *n* = 56) and women with recurrent spontaneous pregnancy loss (RPL, *n* = 23; 2–4 miscarriages). In women following miscarriage, we observed increased frequencies of immature (Figure 2A) and B10-like (Figure 2C) cells. Interestingly, an increase in the frequency of immature B cells was noted in women who experienced one miscarriage (Figure 2B), whereas an increased frequency of B10-like cells was observed in women in the RPL group (Figure 2D). A decreased frequency of plasmablasts with the CD19^−^CD27^int^CD38^+^ phenotype was observed (Figure 2E,F) in women after miscarriage.

### 2.2. Decreased Proportions of IL-35-Producing B10-like Cells and Plasmablasts in Women with RPL

**Figure 3:**
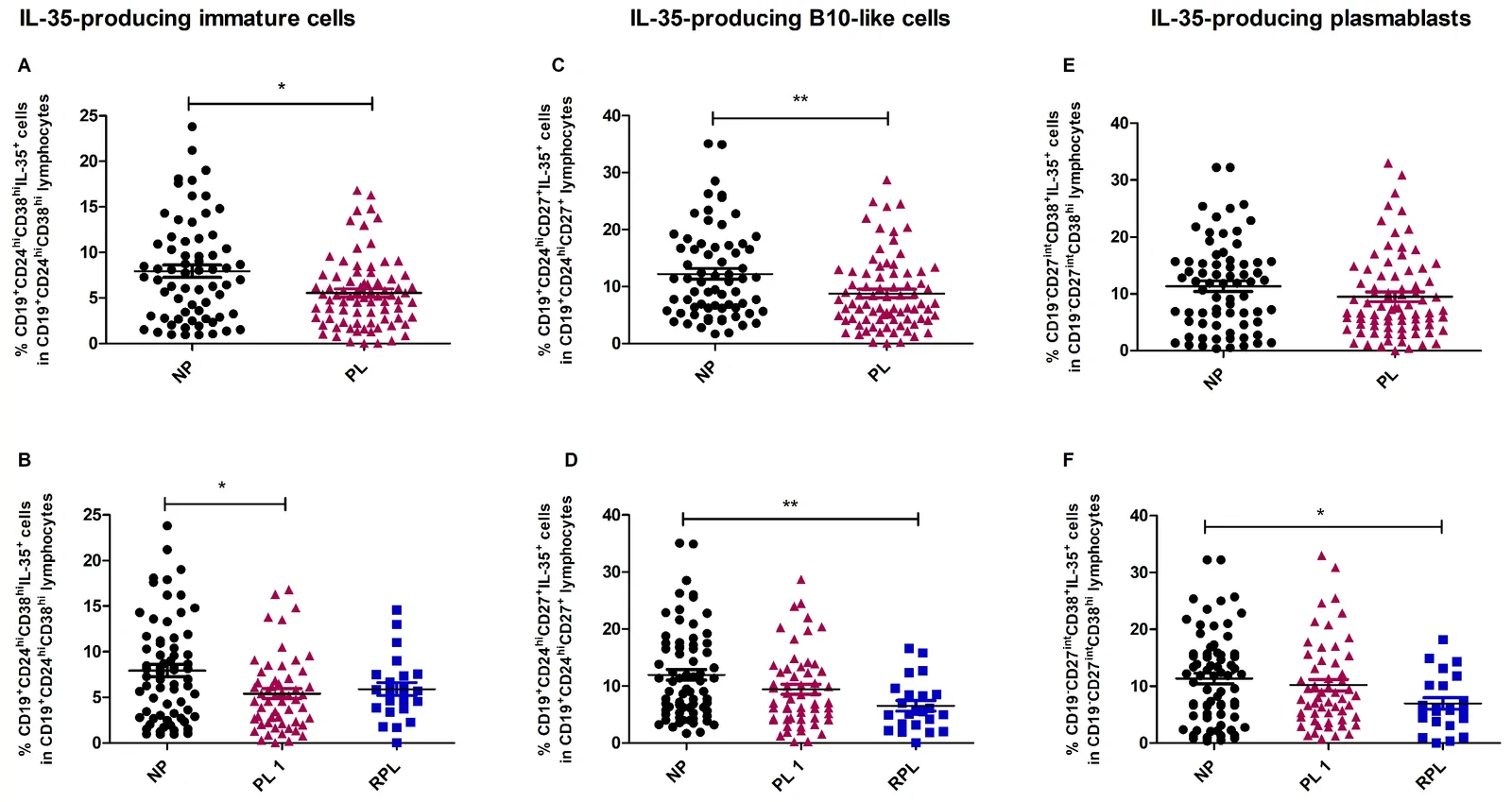
Frequencies of IL-35–producing immature cells (**A**,**B**), B10-like cells (**C**,**D**) and plasmablasts (**E**,**F**) within parent lymphocyte populations in peripheral venous blood from healthy women (NP—normal pregnancy, *n* = 68) and women after miscarriages (PL—pregnancy loss (total), *n* = 79; PL1—group of women who experienced one miscarriage, *n* = 56; RPL—group of women affected by 2–4 miscarriages, *n* = 23). Data are expressed as mean ± standard error of the mean (SEM). Normality was assessed using the Shapiro–Wilk normality or Kolmogorov–Smirnov tests. * *p* < 0.05, ** *p* < 0.01 as analyzed by Student’s *t*-test (parametric) or Mann–Whitney U test (non-parametric) to compare two groups or one-way analysis of variance with Tukey’s multiple comparison test (parametric) or Kruskal–Wallis test with Dunn’s multiple comparison test (non-parametric) to compare three groups.

**Figure 4:**
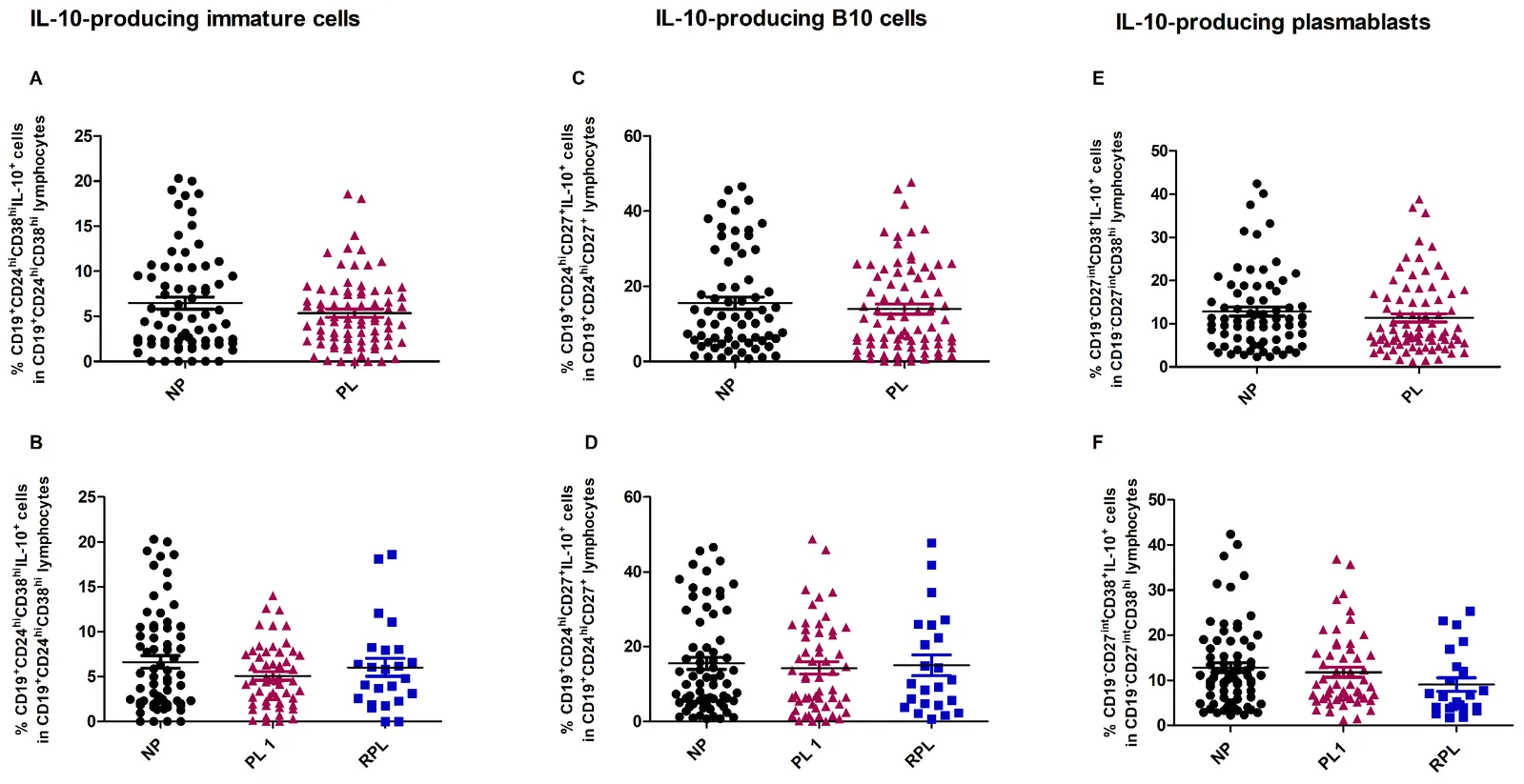
Frequencies of IL-10–producing immature cells (**A**,**B**), B10 cells (**C**,**D**) and plasmablasts (**E**,**F**) within parent lymphocyte populations in peripheral venous blood from healthy women (NP—normal pregnancy, *n* = 68) and women after miscarriages (PL—pregnancy loss (total), *n* = 79; PL1—group of women who experienced one miscarriage, *n* = 56; RPL—group of women affected by 2–4 miscarriages, *n* = 23). Data are expressed as mean ± standard error of the mean (SEM). Normality was assessed using the Shapiro–Wilk normality or Kolmogorov–Smirnov tests. Data were analyzed by Student’s *t*-test (parametric) or Mann–Whitney U test (non-parametric) to compare two groups or one-way analysis of variance with Tukey’s multiple comparison test (parametric) or Kruskal–Wallis test with Dunn’s multiple comparison test (non-parametric) to compare three groups.

In contrast to the subpopulations of B cells presented in Figure 2, opposite trends were observed for IL-35-producing B cells (Figure 3A–D). The proportion of IL-35-producing immature B cells decreased in women after the first miscarriage (Figure 3B), whereas the frequency of IL-35-producing B10-like cells decreased in women with RPL (Figure 3D). Similar to IL-35-producing B10-like cells, the proportion of IL-35-producing plasmablasts also decreased in the RPL group (Figure 3F). No differences were observed in the percentages of IL-10-producing plasmablasts, immature B cells, or B10 cells between the studied female groups (Figure 4A–F).

### 2.3. Decreased PD-L1^+^ B10-like and Immature B Cell Proportions in Women with RPL

**Figure 5:**
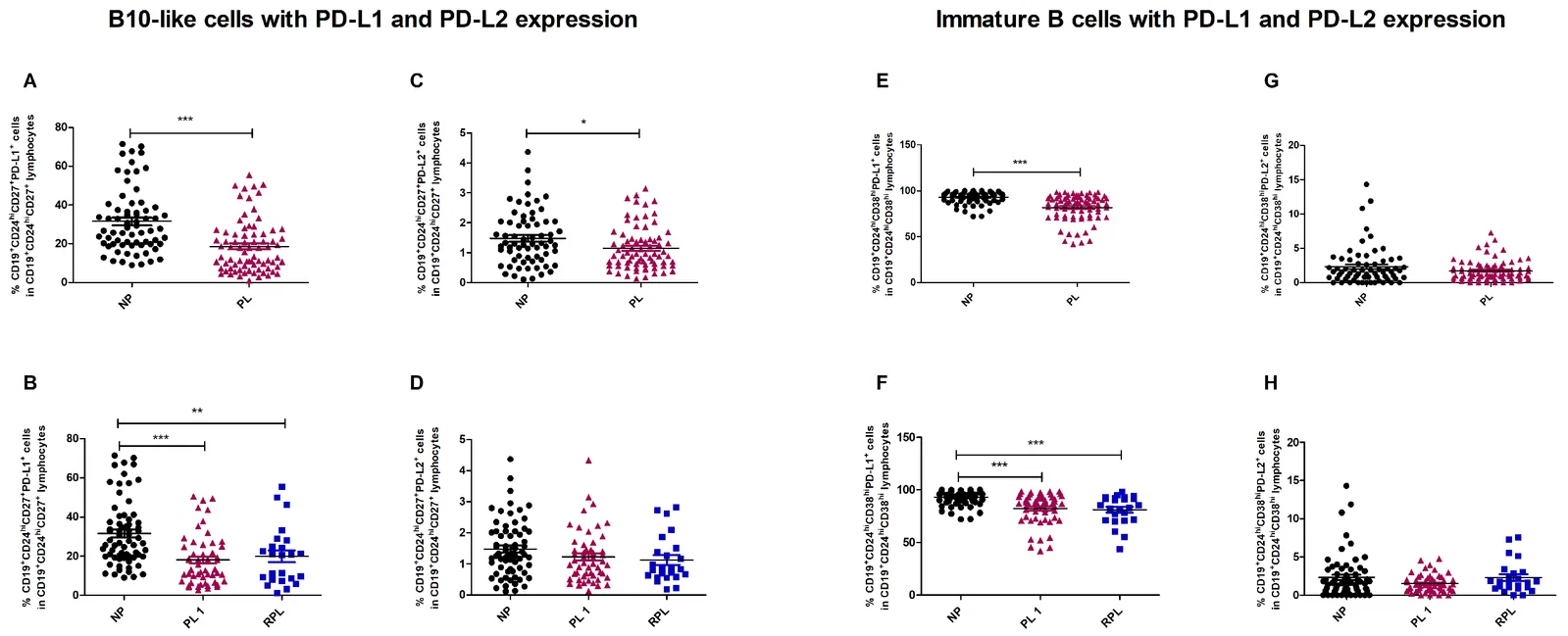
The percentages of B10-like and immature B cells expressing PD-L1 ((**A**,**B**) for B10-like cells and (**E**,**F**) for immature B cells) and PD-L2 expression ((**C**,**D**) for B10-like cells and (**G**,**H**) for immature B cells) within parent lymphocyte populations in peripheral venous blood from healthy women (NP—normal pregnancy, *n* = 68) and women after miscarriages (PL—pregnancy loss (total), *n* = 79; PL1—group of women who experienced one miscarriage, *n* = 56; RPL—group of women affected by 2–4 miscarriages, *n* = 23). Data are expressed as mean ± standard error of the mean (SEM). Normality was assessed using the Shapiro–Wilk normality or Kolmogorov–Smirnov tests. * *p* < 0.05, ** *p* < 0.01, *** *p* < 0.001 as analyzed by Student’s *t*-test (parametric) or Mann–Whitney U test (non-parametric) to compare two groups or one-way analysis of variance with Tukey’s multiple comparison test (parametric) or Kruskal–Wallis test with Dunn’s multiple comparison test (non-parametric) to compare three groups.

We observed decreased frequencies of PD-L1^+^ B10-like and immature B cells within the parent populations in women with RPL (Figure 5B and Figure 5F, respectively). Additionally, the PD-L1^+^ and PD-L2^+^ B10-like cell proportions and PD-L1^+^ immature B cell frequency decreased in the total PL group (Figure 5A, Figure 5C, and Figure 5E, respectively). We did not observe any changes in PD-L2^+^ B10-like and immature B cells in the PL1 and RPL groups (Figure 5D and Figure 5H, respectively), or in PD-L2^+^ immature cells in the total PL group (Figure 5G). Regarding plasmablasts, PD-L2 expression was not detected, whereas no significant differences in PD-L1 expression were observed between the study groups. 

### 2.4. Decreased the IL-35^+^, IL-10^+^, TGF-β^+^ and PD-1^+^ Tregs, as Well as, iTr35, Tr1 and Th3 Cells Frequencies in Women After Miscarriages

**Figure 6:**
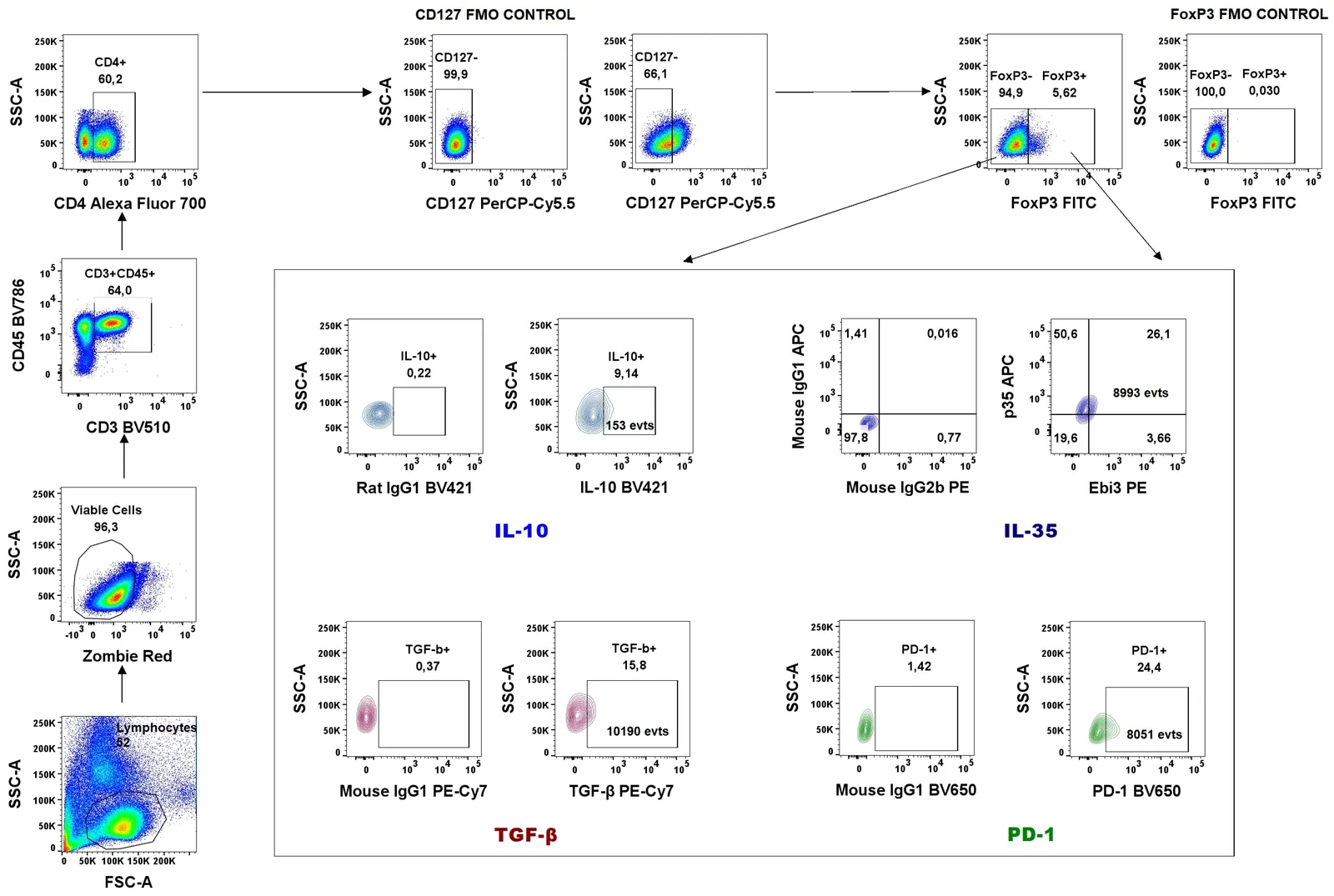
Representative dot-plots showing the gating strategy of Tregs (CD45^+^CD3^+^CD4^+^CD127^−^Foxp3^+^) with IL-35, IL-10, TGF-β and PD-1 expression, as well as iTr35 (CD45^+^CD3^+^CD4^+^CD127^−^Foxp3^−^IL-35^+^), Tr1 (CD45^+^CD3^+^CD4^+^CD127^−^Foxp3^−^IL-10^+^) and Th3 (CD45^+^CD3^+^CD4^+^CD127^−^Foxp3^−^TGF-β^+^) cells. For cytokine analysis and PD-1 molecule, we used appropriate isotype controls in the same concentration as specific antibodies. For other surface antigens, FMO controls were used. Representative flow-cytometry plots illustrating regulatory T cell subpopulations’ resolution in the study groups are presented in Supplementary Data (Figure S2).

**Figure 7:**
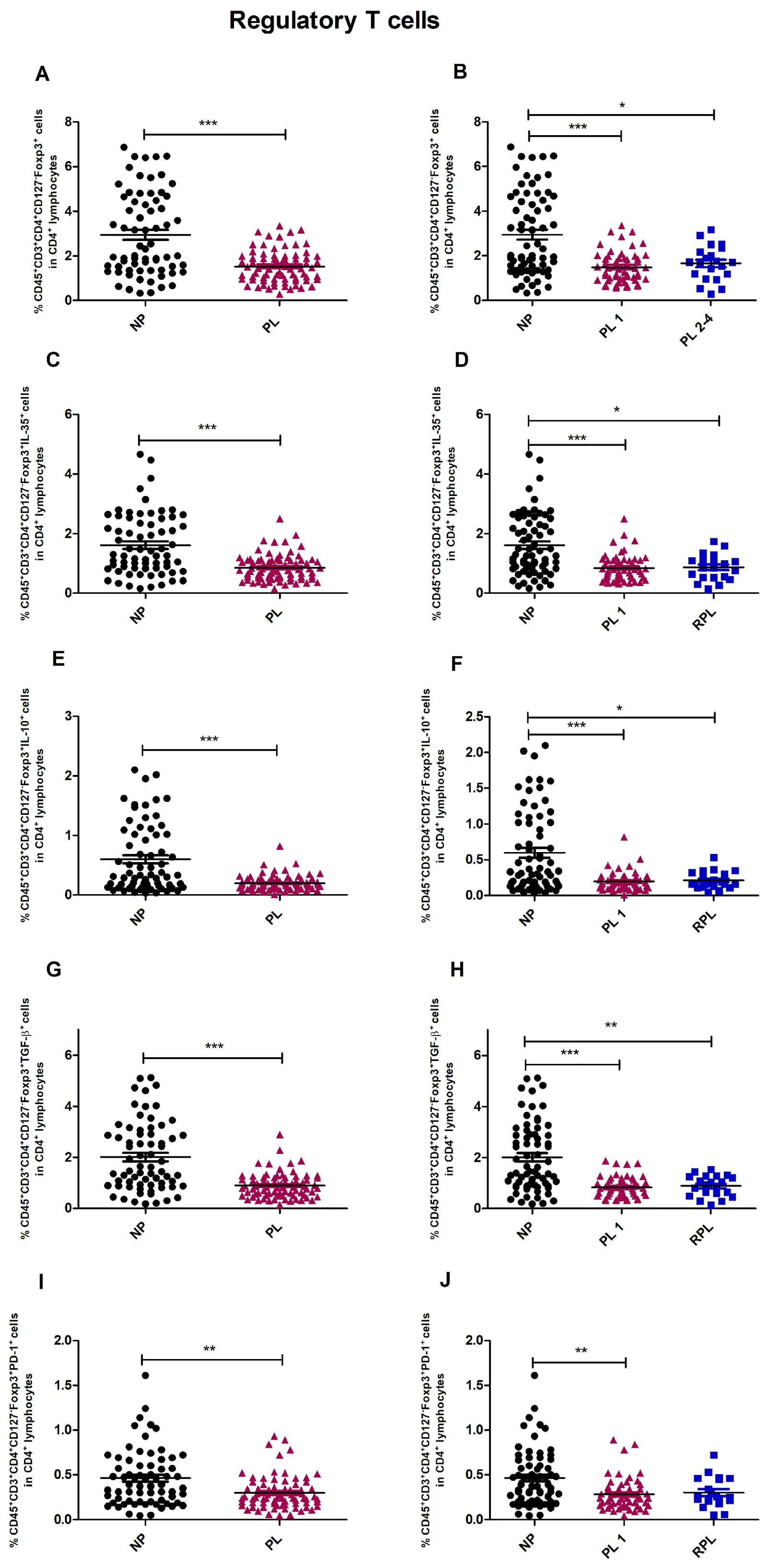
Frequencies of Tregs (CD45^+^CD3^+^CD4^+^CD127^−^Foxp3^+^) (**A**,**B**), IL-35-producing Tregs (CD45^+^CD3^+^CD4^+^CD127^−^Foxp3^+^IL-35^+^) (**C**,**D**), IL-10-producing Tregs (CD45^+^CD3^+^CD4^+^CD127^−^Foxp3^+^IL-10^+^) (**E**,**F**), TGF-β-producing Tregs (CD45^+^CD3^+^CD4^+^CD127^−^Foxp3^+^TGF-β^+^) (**G**,**H**) and Tregs with PD-1 expression (**I**,**J**) within CD4^+^ lymphocytes in peripheral venous blood from healthy women (NP—normal pregnancy, *n* = 68) and women after miscarriages (PL—pregnancy loss (total), *n* = 79; PL1—group of women who experienced one miscarriage, *n* = 56; RPL—group of women affected by 2–4 miscarriages, *n* = 23). Data are expressed as mean ± standard error of the mean (SEM). Normality was assessed using the Shapiro–Wilk normality or Kolmogorov–Smirnov tests. * *p* < 0.05, ** *p* < 0.01, *** *p* < 0.001 as analyzed by Student’s *t*-test (parametric) or Mann–Whitney U test (non-parametric) to compare two groups or one-way analysis of variance with Tukey’s multiple comparison test (parametric) or Kruskal–Wallis test with Dunn’s multiple comparison test (non-parametric) to compare three groups.

**Figure 8:**
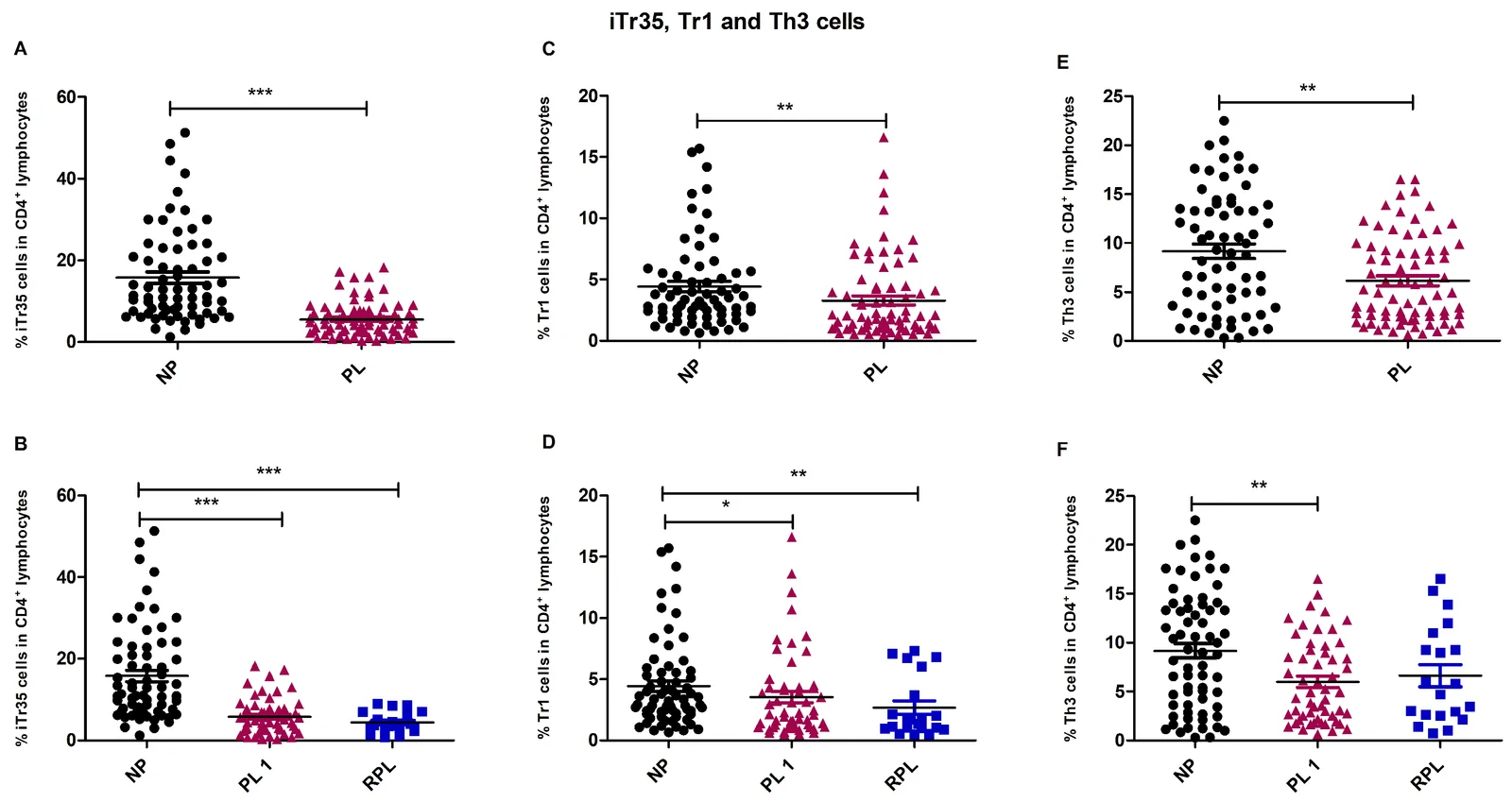
The proportions of iTr35 (CD45^+^CD3^+^CD4^+^CD127^−^Foxp3^−^IL-35^+^) (**A**,**B**), Tr1 (CD45^+^CD3^+^CD4^+^CD127^−^Foxp3^−^IL-10^+^) (**C**,**D**) and Th3 (CD45^+^CD3^+^CD4^+^CD127^−^Foxp3^−^TGF-β^+^) (**E**,**F**) cells within CD4^+^ lymphocytes in peripheral venous blood from healthy women (NP—normal pregnancy, *n* = 68) and women after miscarriages (PL—pregnancy loss (total), *n* = 79; PL1—group of women who experienced one miscarriage, *n* = 56; RPL—group of women affected by 2–4 miscarriages, *n* = 23). Data are expressed as mean ± standard error of the mean (SEM). Normality was assessed using the Shapiro–Wilk normality or Kolmogorov–Smirnov tests. * *p* < 0.05, ** *p* < 0.01, *** *p* < 0.001 as analyzed by Student’s *t*-test (parametric) or Mann–Whitney U test (non-parametric) to compare two groups or one-way analysis of variance with Tukey’s multiple comparison test (parametric) or Kruskal–Wallis test with Dunn’s multiple comparison test (non-parametric) to compare three groups.

In the next step, the proportions of Tregs and Tregs producing immunosuppressive cytokines—IL-35, IL-10 and TGF-β were examined based on the gating strategy shown in Figure 6. Regardless of the number of miscarriages, in women after miscarriage we observed a decrease in the frequency of Tregs (Figure 7A,B) and Tregs producing IL-35 (Figure 7C,D), IL-10 (Figure 7E,F) and TGF-β (Figure 7G,H). Additionally in PL group, especially in PL1 group decreased the PD-1^+^ Tregs proportion was noted (Figure 7I,J). The frequency of regulatory cells—iTr35, Tr1 and Th3 was also lower in women after miscarriage (Figure 8A–F).

### 2.5. PD-L1^+^ B10-like Cells Are Negatively Correlated with Tregs, IL-35^+^ Tregs, IL-10^+^ Tregs, TGF-β^+^ Tregs, and Th3 Cells in Normal Pregnancy

**Figure 9:**
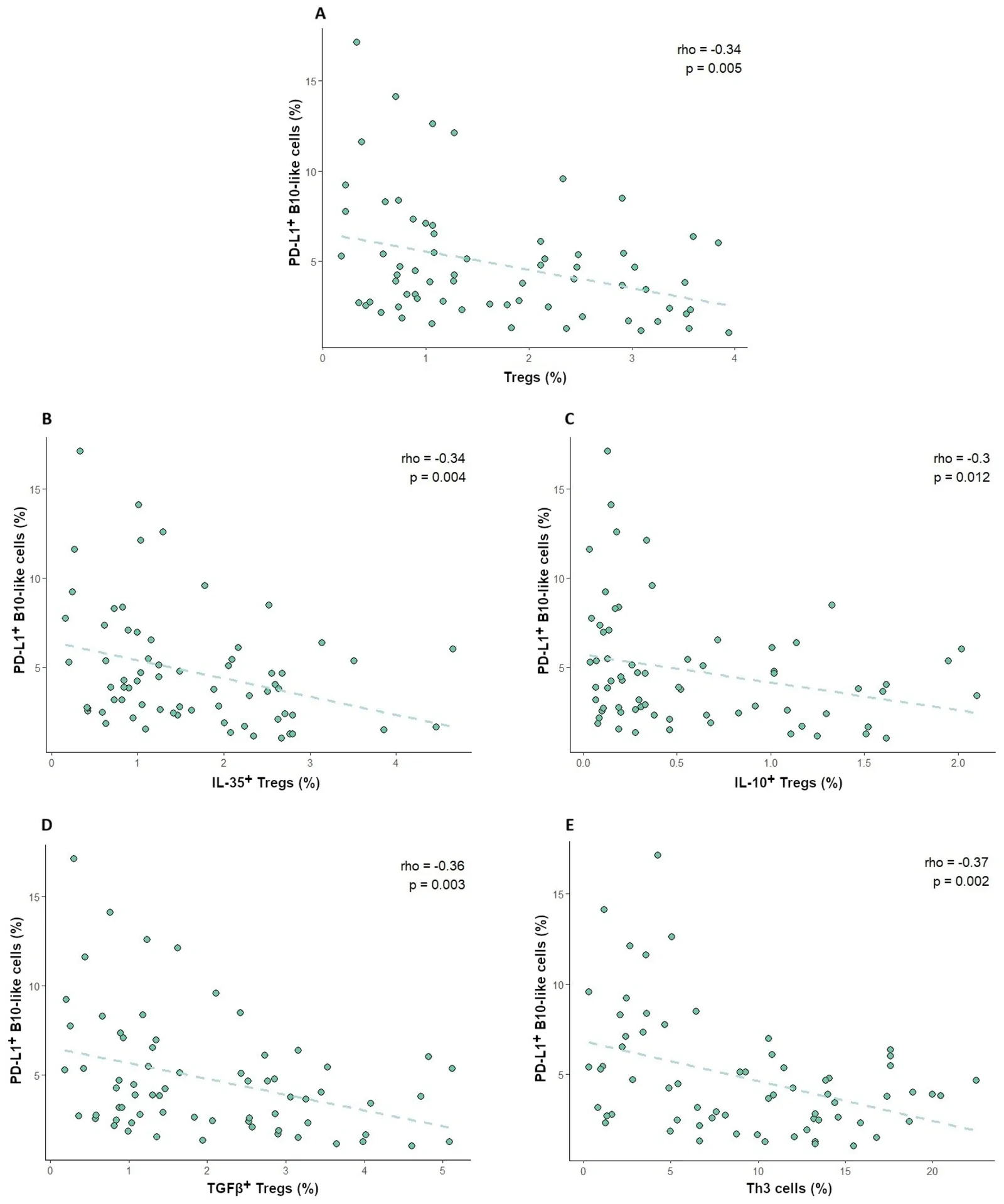
Breg associations with Tregs and Th3 cells. The percentage of PD-L1^+^ B10-like cells (within CD19^+^ cells) in each participant was matched to the respective Treg cell subsets (within CD4^+^ cells) (total Tregs (**A**), IL-35^+^ (**B**), IL-10^+^ (**C**), TGF-β^+^ (**D**)) and to Th3 cells (**E**). Spearman’s correlation analysis was performed to assess the correlations between Bregs and Tregs. The dashed lines represent the linear regression analysis with corresponding rho (correlation coefficient) values and *p*-values; *p* < 0.05 was considered statistically significant.

In normal pregnancy, we observed negative correlations (Figure 9A–E) between the frequency of PD-L1^+^ B10-like cells and the total Treg population (Figure 9A), IL-35^+^ Tregs (Figure 9B), IL-10^+^ Tregs (Figure 9C), TGF-β^+^ Tregs (Figure 9D), and Th3 cell frequency (Figure 9E). These correlations were limited to the normal pregnancy group and were not observed in the PL1 and RPL groups (*p* > 0.05). To further assess differences in correlation strength between groups, correlation coefficients were compared using Fisher’s z-transformation; the corresponding statistical results are presented in Table 1. The negative correlations observed in NP were significantly stronger than those in PL for Tregs, IL-35^+^ Tregs, IL-10^+^ Tregs, and TGF-β^+^ Tregs, whereas no significant differences were observed between NP and RPL for these associations. For Th3 cells, the correlation strength did not differ between NP and PL, as well as NP and RPL groups.

### 2.6. iTr35 Cells Are Positively Correlated with IL-35-Producing Regulatory B Cells in Normal Pregnancy

**Figure 10:**
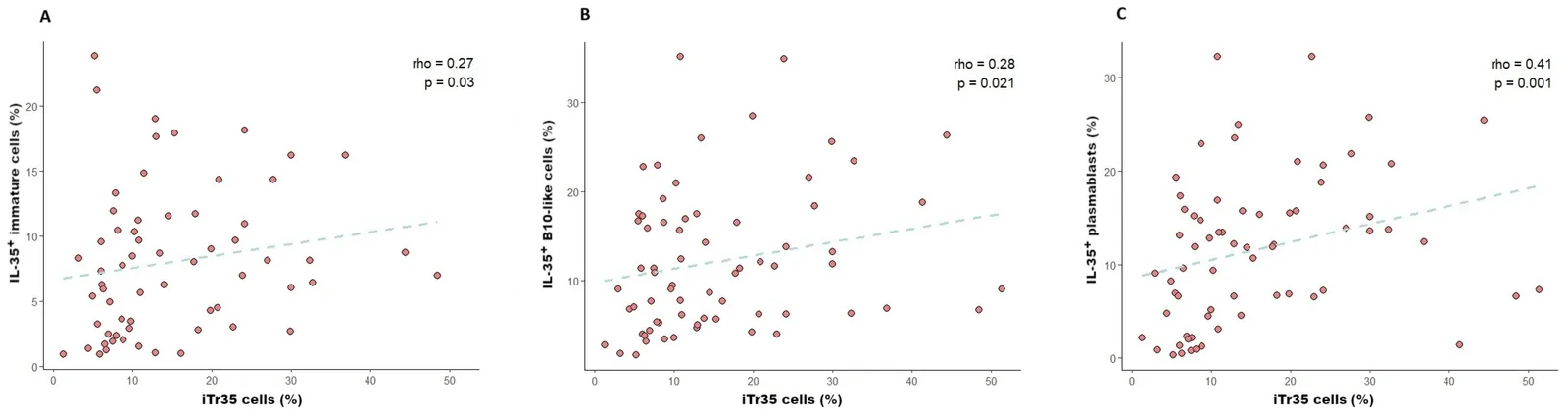
Correlation between % IL-35^+^ immature cells (**A**), % IL-35^+^ B10-like cells (**B**), % IL-35^+^ plasmablasts (**C**), and % iTr35 cells. The dashed lines represent the linear regression analysis with corresponding rho (correlation coefficient) values and *p*-values; *p* < 0.05 was considered statistically significant.

**Figure 11:**
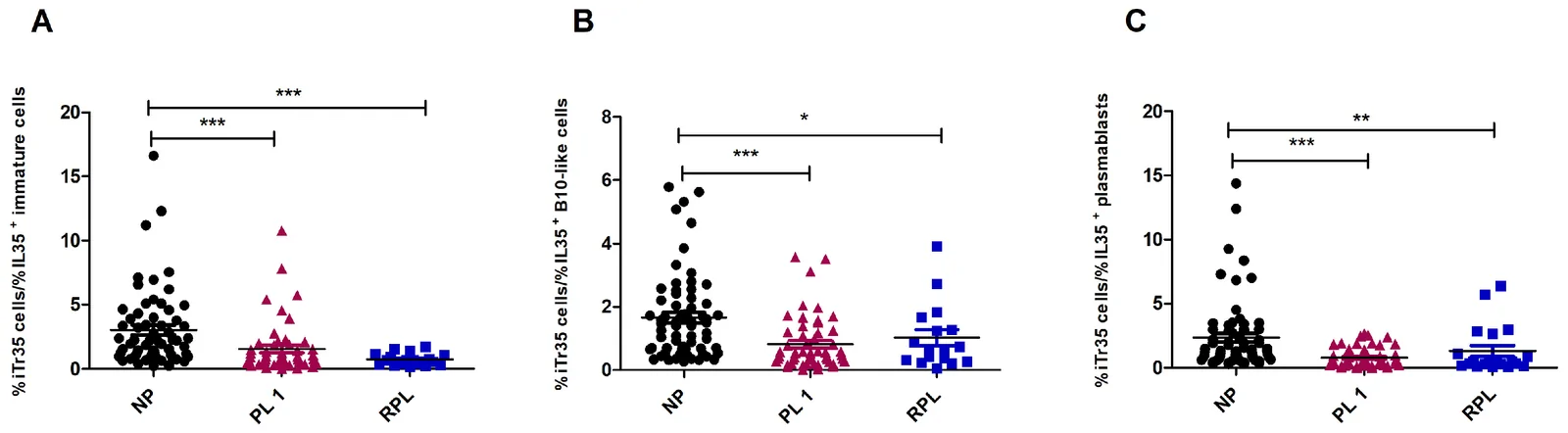
The % iTr35/% IL-35^+^ immature cells (**A**), % iTr35/% IL-35^+^ B10-like cells (**B**) and % iTr35/% IL-35^+^ plasmablasts (**C**) ratios. Data are expressed as mean ± standard error of the mean (SEM). Normality was assessed using the Shapiro–Wilk normality or Kolmogorov–Smirnov tests. * *p* < 0.05, ** *p* < 0.01, *** *p* < 0.001 as analyzed by the Kruskal–Wallis test with Dunn’s multiple comparison test.

**Figure 12:**
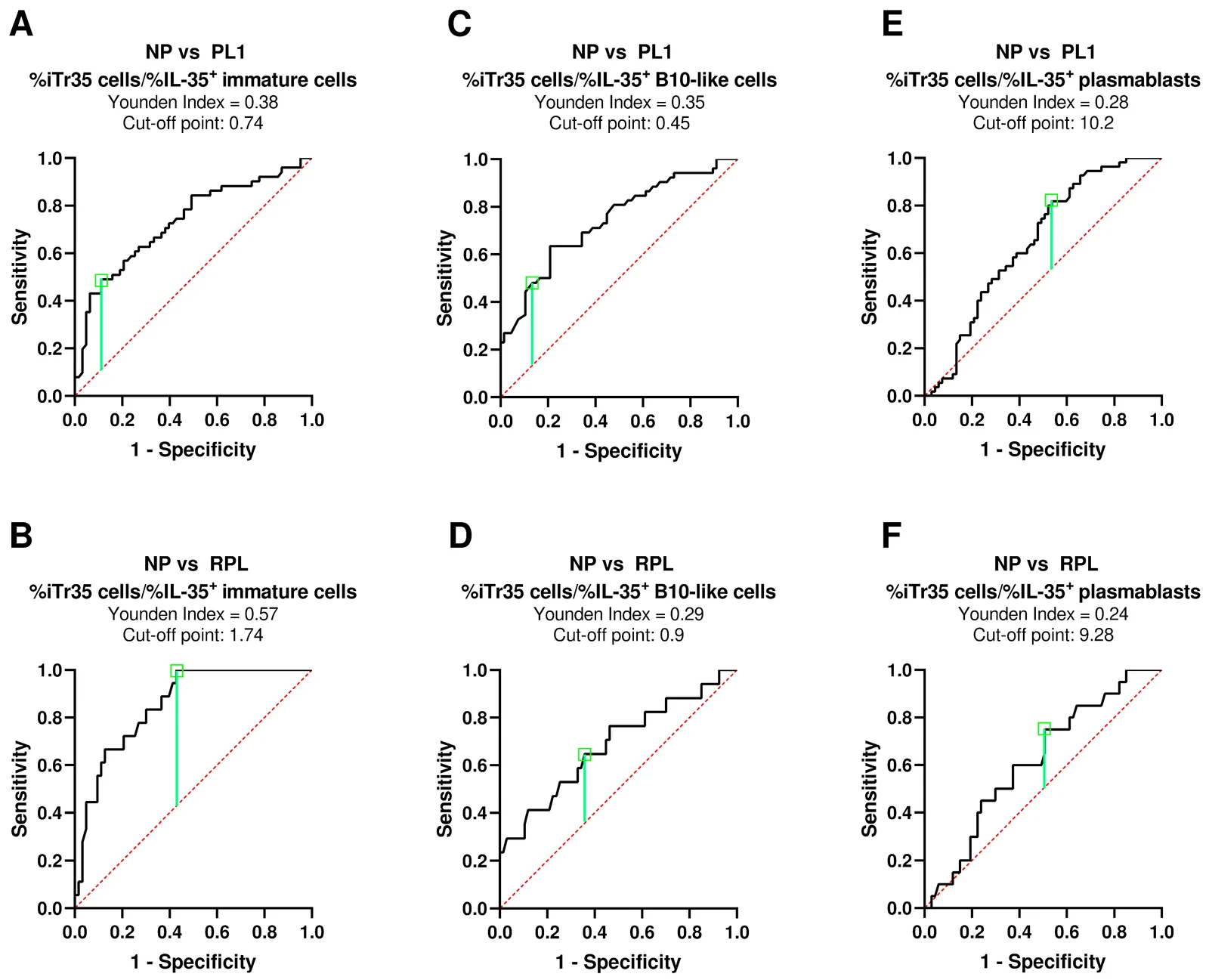
ROC curve analysis of the % iTr35/% IL-35^+^ cells ratios for discrimination between NP, PL1, and RPL groups. % iTr35/% IL-35^+^ immature cells ratio in NP vs. PL1 (**A**) and NP vs. RPL groups (**B**); % iTr35/% IL-35^+^ B10-like cells ratio in NP vs. PL1 (**C**) and NP vs. RPL (**D**) groups; % iTr35/% IL-35^+^ plasmablasts ratio in NP vs. PL1 (**E**) and NP vs. RPL groups (**F**). The Youden index and corresponding optimal cut-off values are presented for each comparison, with the optimal cut-off indicated by a green line.

In normal pregnancy, we also observed positive correlations between % iTr35 cells and % IL-35^+^ regulatory B cell frequencies (B10-like, immature B cells, and plasmablasts) (Figure 10A–C). This result was observed only in healthy pregnancies. In the PL1 and RPL groups, we did not observe any positive correlations (*p* > 0.05). To further determine whether the strength of these associations differed between pregnancy outcome groups, Spearman correlation coefficients were compared using Fisher’s z-transformation. This analysis revealed significant differences in the correlations between iTr35 cells and IL-35^+^ B10-like cells between NP and RPL, as well as between iTr35 cells and IL-35^+^ plasmablasts between NP and both PL and RPL (Table 1). No significant differences were observed for IL-35^+^ immature cells. These findings suggest that the coordinated relationship between IL-35-producing regulatory B cells and iTr35 cells observed during normal pregnancy is altered following pregnancy loss. Next, we assessed the ratios of % iTr35 cells to % IL-35^+^ immature B cells, B10-like cells, and plasmablasts (Figure 11A–C). All three ratios were significantly decreased in the PL1 and RPL groups compared with healthy pregnancies. ROC analysis demonstrated significant discriminatory potential of these ratios between healthy pregnancies and pregnancy loss (Figure 12, Table 2). The strongest discriminatory performance was observed for the % iTr35/% IL-35^+^ immature B cells ratio in NP versus RPL (AUC = 0.8532), suggesting that this ratio may have potential as a candidate biomarker associated with RPL.

## 3. Discussion

Regardless of the cause, miscarriage is a slow inflammatory process that leads to the loss of contact between the mother and fetus and the expulsion of fetal tissue. This process is accompanied by local and systemic hormonal changes (reduction in hCG and progesterone levels), local tissue repair processes leading to restoration of the menstrual cycle, and a shift in the immune response from anti-inflammatory to pro-inflammatory. Since the maintenance of pregnancy is closely linked to the development of maternal immune tolerance, immune cells adapt their phenotype to the changing antigenic environment associated with the presence of an allogeneic fetus. These changes were observed in the periphery. Miscarriage is also accompanied by a change in the phenotype of immune cells, both locally and peripherally [5]. Assessment of the frequency of regulatory cells in the peripheral blood of women after a miscarriage compared to women with a normal pregnancy may be important for investigating the causes of pregnancy loss due to immunological disorders (loss of tolerance), assessing the role of regulatory cells in the process of restoring immune balance, assessing the immunological status of women during pregnancy, and monitoring the state of tolerance accompanying it.

In this study, we aimed to systematize our understanding of the significance of the presence various phenotypes of peripheral blood regulatory cells in normal pregnancy, miscarriages, and recurrent spontaneous miscarriages. We carefully characterized cell populations that have not yet been studied in the context of pregnancy and miscarriage. The cells studied included regulatory B cells, B10-like cells, immature cells, and plasmablasts, in association with the production of immunosuppressive cytokines: IL-10, TGF-beta, and especially IL-35, which is one of the least studied cytokines in pregnancy and miscarriage. Another important aspect was the association of Bregs with the expression of checkpoint molecules PD-L1 and PD-L2, together with a detailed characterization of regulatory T cells producing the aforementioned cytokines and expressing PD-1. Furthermore, these correlations expand our knowledge of the interrelationships between peripheral regulatory cells during normal pregnancy and recurrent spontaneous miscarriages. Additionally, our results highlighted that IL-35-associated regulatory populations may participate in a coordinated immunoregulatory network that could contribute to pregnancy tolerance. It is likely that the appropriate ratio of one regulatory population expressing IL-35 (iTr35) to another IL-35-producing cell—B10-like cells, immature B cells, and plasmablasts—is important for pregnancy success. The strongest discriminatory performance was observed for the % iTr35/% IL-35^+^ immature B cells ratio in NP versus RPL.

Compared with other immune cell subsets, much less is known about the role of B cells in pregnancy. The different B cell phenotypes are profoundly influenced by the external milieu and the presence of foreign antigens [43]. In this study, we observed an increase in the frequency of immature and B10-like cells in women after miscarriage. Interestingly, an increased frequency of immature B cells was noted in women who experienced one miscarriage, whereas an increase in the frequency of B10-like cells was noted in women in the RPL group. This observation may be considered in the broader context of alterations in the CD27^+^ B-cell compartment reported in RPL. In particular, an increased proportion of unswitched memory B cells (CD19^+^CD27^+^IgD^+^) has been described in women with recurrent pregnancy loss [44]. The frequency of CD19^+^CD24^hi^CD27^+^ cells was also examined by Ana Zenclussen’s research team [45]. In contrast to our findings, they reported significantly lower percentages of CD19^+^CD24^hi^CD27^+^ Breg cells in women experiencing miscarriage compared with women with normal pregnancies in the first trimester. These differences were likely due to the small sample size of that study (*n* = 8 pregnant women; *n* = 5 women after miscarriage) and methodological differences between studies. Following miscarriage, the decidua undergoes a pronounced inflammatory response [46], which may initiate processes associated with tissue repair. In this context, B cells are of particular interest, as purified, mature, naïve B220^+^CD19^+^IgM^+^IgD^+^ B cells have been confirmed to significantly improve healing of acute and diabetic wounds in mice following a single topical application [47]. Although these findings suggest that B cells may have a role in tissue repair, their relevance to the post-miscarriage setting remains unclear. Thus, the observed increase in the overall B cell population after miscarriage could potentially be related to processes occurring during tissue recovery. However, this hypothesis warrants further investigation. In the next step of our study, a more detailed characterization of regulatory B cells revealed a significantly reduced percentage of IL-35-producing immature cells in women following their first miscarriage and IL-35-producing B10-like cells in the RPL group. In our previous work, we indicated that the early stages of abortion-prone pregnancy in mice were also characterized by a diminished frequency of B cells producing IL-35 at both the local and peripheral levels [32]. In turn, our recent work shows that IL-35 administration before implantation to abortion-prone mice (CBA/J♀ × DBA/2J♂), increased the frequency of IL-35-producing B10 cells and IL-35-producing Tregs at the periphery, as well as CD19^+^IL-35^+^ B cells in decidua [41]. These results indicated the potential involvement of IL-35 in the establishment of tolerance during pregnancy through its multifaceted effects on regulatory cells. The findings from another research group [39] have shown that IL-35 promotes the development of IL-35-producing Breg cells and enhances the expansion of B10 cells, also supporting a functional association between IL-35 and Breg-mediated immune regulation during pregnancy. Interestingly, in the case of IL-10-producing immature cells, B10 cells, and plasmablasts, no differences were observed between women whose pregnancies were normal and those who experienced miscarriage. Taken together, the selective reduction in IL-35-producing B10-like cells, in the absence of changes in IL-10-producing B10 cells, may suggest a selective alteration of the functional profile of the B10-like compartment rather than a generalized loss of regulatory capacity. The reason for this difference in anti-inflammatory cytokine expression remains unclear. Nevertheless, in more advanced stages of pregnancy and preterm birth, lower percentages of IL-10-producing B10 cells and IL-10-producing immature B cells were observed [48,49]. Without fetal karyotyping, our results can only provide additional information about the inflammatory processes occurring at the time of miscarriage, but cannot provide any information on whether the visible changes are the cause of the miscarriage. Undoubtedly, the changes observed in normal pregnancy compared to the inflammation accompanying miscarriage may contribute to the success of pregnancy, and future studies investigating IL-35 expression dynamics may provide important insights.

Another aspect of the study that we focused on was the PD-1/PD-L1 and PD-L2 pathways. Zhang et al. [23] demonstrated the critical function of the PD-1/PD-L1 pathway in regulating T cell homeostasis and peripheral tolerance by promoting the development and function of Tregs and inhibiting the activation of effector T cells [23]. PD-L1 blockade in pregnant mice increased the number of abortions of allogeneic fetuses, enhanced the expansion of Th1 cells in vivo [50,51], decreased the number of Tregs, and increased Th17 proportion, suggesting a critical impact of the PD-1/PD-L1 pathway on the immune balance among Th1, Th2, Th17, and Tregs [52], which is crucial for successful pregnancy. Additionally, PD-L2 blockade in allogeneic murine pregnancies did not affect fetal resorption [50]. PD-L2, like PD-L1, is present in the placenta throughout pregnancy but may have a weaker tolerogenic function than PD-L1 [30]. We observed decreased PD-L1^+^ and PD-L2^+^ B10-like cell proportions in women after miscarriages, as well as a decreased frequency of PD-L1^+^ immature B cells in the same study group. Additionally, in the PL group, particularly in the PL1 subgroup, a decreased proportion of PD-1^+^ Tregs was noted. The significant reduction in PD-1^+^ Tregs observed only in the PL1, but not RPL group may have several possible explanations, including biological differences between women experiencing a single pregnancy loss and those with recurrent losses, as well as the limited sample size of the RPL group. Given the cross-sectional design of our study, however, we cannot determine the underlying reason for this subgroup difference. In Wang research [53], the proportion of PD-1^+^ Tregs (CD4^+^CD25^+^CD127^dim/−^CD279^+^) in the RPL group was not different from that in controls. However, the authors observed that PD-1 and PD-L1 expression in Th17 cells and PD-1 expression in Th1 cells were significantly downregulated in women with RPL. This may lead to an increase in Th1 and Th17 responses and an imbalance among Th17, Th1, and Treg cells in women with RPL. Our findings suggest that the observed changes in peripheral regulatory B and T cell populations may be relevant to immune regulation during healthy pregnancy.

Many studies have reported that Tregs play a key role in maintaining pregnancy, and their dysregulation may contribute to obstetric complications, such as miscarriage, preterm birth, or gestational diabetes [54]. In women with unexplained RPL, the proportion of CD4^+^ Tregs in the peripheral blood and decidua was lower than that in healthy pregnant women [6,55]. Moreover, adoptive transfer of Tregs reversed the increased abortion rate in an abortion-prone mouse model [18,56]. Our study confirmed that the proportion of Tregs in the peripheral blood was lower in women with unexplained RPL than in healthy pregnant women. Our results expands the characterization of the Treg phenotype, including the cytokines they produce, important for establishing immunotolerance: IL-10, TGF-β, and especially the least studied IL-35. This result is consistent with our previous findings and highlights the importance of IL-35 in the success of pregnancy [41]. Our study shows for the first time that women with normal pregnancy have a higher percentage of iTr35 cells than women experiencing miscarriage. This novel subpopulation of Tregs is characterized by phenotypic stability, lack of Foxp3 expression, and a potent immunosuppressive function through IL-35 production [34]. Similarly to iTr35 cells, increased frequencies of IL-10-producing Tr1 cells and TGF-β-producing Th3 cells were also observed in the peripheral blood of women with normal pregnancies compared to women who had experienced miscarriages.

Next, we investigated the correlations between Breg and Treg cells, as well as between different populations of regulatory cells producing IL-35. Since Breg and Treg cells occur in close proximity to the human decidua, forming clusters that suggest functional interactions [19], we speculated that the situation might be similar in the periphery. Additionally, we noted a unique feature of IL-35, namely its role in “infectious tolerance”. IL-35 induces Breg cells and promotes their conversion into a subset of Breg cells that also begin to produce IL-35. The negative correlation between PD-L1^+^ B10-like cells and Tregs observed by our team confirmed and complemented recent studies by Wiley [57], who showed that PD-L1^+^ Bregs were negatively correlated with Tregs and Helios^+^, CTLA-4^+^, PD-1^+^, TIGIT^+^, and TIM3^+^ Tregs. We, in turn, observed negative correlations between PD-L1^+^ Bregs and Tregs, including IL-35^+^, IL-10^+^, and TGF-β^+^ Treg subsets, as well as Th3 cells. Interestingly, these correlations were observed only in women with normal pregnancies and were absent in women who had miscarried, both when analyzed as a whole and in the RPL group. The inverse correlations between PD-L1^+^ B10-like cells and Treg subsets may reflect several, not mutually exclusive, mechanisms. They may represent a compensatory relationship between different regulatory populations, in which increased involvement of one regulatory subset is accompanied by reduced representation of another. Alternatively, these associations may reflect differences in the activation or functional state of these cells. The absence of these correlations in women with pregnancy loss may also indicate an altered organization of the regulatory network associated with pregnancy loss. However, given the cross-sectional nature of our study, temporal changes following pregnancy loss cannot be assessed. The interplay between Tregs and Bregs may therefore represent an important component of the regulatory network supporting immune tolerance during pregnancy. The PD-1/PD-L1 pathway is involved in the regulation of T cell homeostasis and peripheral tolerance by promoting the development and function of Treg cells and inhibiting the activation of effector T cells. Additionally, the immunoregulatory function of Tregs associated with the production of immunosuppressive cytokines may occur one after the other and complement each other. Interestingly, our research indicated a positive correlation between IL-35-producing immature cells, B10-like cells, plasmablasts, and the iTr35 population in the peripheral blood of women with normal pregnancies. These findings were limited to healthy pregnancies and were not observed in miscarriages. Although these correlations may be consistent with functional interactions or coordinated regulation between these cell populations, they may also reflect a shared biological or physiological factors associated with pregnancy rather than direct cellular interactions. Therefore, the observed associations should be interpreted cautiously. Given the proposed role of IL-35 in the induction and propagation of regulatory responses, these findings may be consistent with the concept of IL-35-mediated infectious tolerance described previously [37]. The proportions of iTr35/IL-35^+^ immature cells, iTr35/IL-35^+^ B10-like cells, and iTr35/IL-35^+^ plasmablasts were 2–4 times higher in normal pregnancies than after miscarriage. The interrelationships between distinct peripheral Breg and Treg subpopulations may be involved in the complex immunoregulatory network associated with immune tolerance during pregnancy and may be relevant to the prevention of obstetric complications. The loss of positive and negative correlations between Tregs and Bregs observed after miscarriage may indicate that inflammation accompanying pregnancy loss disrupts the tightly regulated interactions between these two cell populations during pregnancy. Moreover, the differences in iTr35/IL-35^+^ B-cell ratios and their discriminatory potential suggest that these ratios, particularly the % iTr35/% IL-35^+^ immature B cells ratio, may have potential as a candidate biomarker associated with RPL.

Despite the novel contribution of our study to the understanding of maternal immune tolerance, several limitations should be considered when interpreting our findings. First, the cross-sectional and descriptive design, together with the collection of samples after pregnancy loss, precludes determination of whether the observed immune alterations contributed to pregnancy failure or resulted from the inflammatory and tissue-remodeling processes accompanying miscarriage. Although the cross-sectional design does not allow us to determine whether the observed differences preceded or resulted from pregnancy loss, the higher levels of IL-35-related regulatory parameters observed during normal pregnancy are consistent with a distinct regulatory immune profile compared with pregnancy loss. Second, the analysis of peripheral blood rather than decidual tissue limits the extent to which our findings can be extrapolated to immune processes at the maternal-fetal interface. Third, the absence of functional assays limits mechanistic interpretation, and such studies are planned for future research. The relatively small size of the RPL subgroup should also be considered when interpreting subgroup-specific findings. Moreover, comprehensive diagnostic evaluation of recognized causes of RPL, including antiphospholipid syndrome, inherited thrombophilia, parental or embryonic/fetal chromosomal abnormalities, and endocrine, or other recognized causes, was not performed. Such evaluation would be required to classify cases as unexplained recurrent pregnancy loss (uRPL). Therefore, our cohort should not be considered a population with uRPL, but rather as women with recurrent spontaneous pregnancy loss (RPL) of unknown or uninvestigated etiology. The lack of unstimulated and internal non-regulatory controls should also be considered when interpreting changes observed following PMA/ionomycin stimulation. Although PMA/ionomycin stimulation may affect surface marker expression, the identical stimulation conditions applied to all study groups minimized the potential confounding effect of the stimulation procedure on between group comparisons. Another limitation of the present study is the analysis of relatively rare cell populations. This was particularly apparent for PD-L2-positive populations, for which fewer than 100 events were detected in the terminal gate (Figure S1). Accordingly, the results obtained for these low-frequency populations should be interpreted with caution. Finally, IL-35-producing cells were identified by intracellular co-expression of Ebi3 and p35, which does not directly demonstrate assembly and secretion of the functional IL-35 heterodimer.

In summary, in a group of women with recurrent spontaneous miscarriages of uninvestigated etiology, we observed an increased percentage of B10-like cells with a CD19^+^CD24^hi^CD27^+^ phenotype and plasmablasts (CD19^−^CD27^int^CD38^+^), which may be related to tissue regeneration after miscarriage and warrants further investigation. Inflammation associated with miscarriage is characterized by a decreased proportion of IL-35-producing regulatory cells, including immature B cells, B10-like cells, plasmablasts, Tregs, and iTr35 cells. Here, we also revealed that the frequency of PD-L1-expressing B10-like cells was negatively correlated with the frequency of Tregs and Th3 cells, suggesting that the interplay between Bregs and Tregs may be part of a coordinated immunoregulatory network associated with immune tolerance during pregnancy. For the first time, in healthy pregnancies, we observed a positive correlation between IL-35-producing Bregs and iTr35 cell frequencies, and iTr35/IL-35^+^ Breg ratios were 2–4 times higher than those after miscarriage. Therefore, these ratios showed potential discriminatory value between healthy pregnancy and pregnancy loss groups and may warrant further investigation as candidate markers in prospective studies. The strongest discriminatory performance was observed for the % iTr35/% IL-35^+^ immature B cells ratio in NP versus RPL. Together with findings from our previous studies, these results support a potential role of IL-35-associated regulatory mechanisms in immune regulation during pregnancy. Further research, particularly functional and prospective studies, is needed to clarify the relationships among regulatory cell populations and their relevance to normal pregnancy and its complications.

## 4. Materials and Methods

### 4.1. Patients

The study protocol was approved by the Bioethical Commission of the Medical University of Wrocław (no. KB-586A/2020 and KB-984/2021). Each patient signed an informed consent form to participate in the study and process their personal data for research purposes. Peripheral venous blood from healthy pregnant women (NP, *n* = 68) and women with inevitable miscarriage, during bleeding (*n* = 79 for pregnancy loss (PL, total); *n* = 56 for the group of women who experienced one miscarriage (PL1); *n* = 23 for women with recurrent spontaneous pregnancy loss (RPL), defined as a history of 2–4 spontaneous miscarriages) in first trimester of pregnancy were collected from the Gynecology and Obstetrics Department of the Falkiewicz Hospital in Wrocław and Healthy Woman Medical Center in Wrocław. Patients aged 18–41 came from Wrocław and its surrounding areas. The normal pregnancy (NP) group represented women with an ongoing pregnancy at the time of sampling. Gestational age was calculated based on the date of the last menstrual period and ultrasound-based gestational date. Women experiencing pregnancy loss were recruited based on medical history and clinical examination confirming vaginal bleeding and fetal demise (complete lack of heartbeat). Blood samples were collected on the day of diagnosis of pregnancy loss, prior to uterine evacuation and administration of any premedication. Samples were processed within 1–2 h of collection. Patients with symptoms of a cold, hypertension (preeclampsia in previous pregnancies), anatomical defects of the uterus, diabetes, hypothyroidism, or other autoimmune diseases were excluded. Patients who gave birth to a child with a genetic defect or who had a close family member with such a disease, smoked cigarettes, or drank alcohol during pregnancy were also excluded. Although women in the RPL group had a history of 2–4 spontaneous miscarriages, a systematic diagnostic evaluation for all established causes of recurrent pregnancy loss was not performed. In particular, antiphospholipid syndrome, inherited thrombophilia, parental chromosomal abnormalities, embryonic/fetal chromosomal abnormalities, and anatomical, endocrine, or other recognized causes of RPL were not systematically assessed. Therefore, the RPL group should not be considered a cohort with unexplained recurrent pregnancy loss (uRPL), and only recurrent spontaneous pregnancy loss (RPL). Therefore, our RPL cohort represents recurrent spontaneous pregnancy loss (RPL) of uninvestigated etiology rather than unexplained recurrent pregnancy loss (uRPL). For each woman, body mass index (BMI) was calculated. The patient characteristics are shown in Table 3. No differences were found in patients’ age, BMI, gestational age, or number of pregnancies (*p* > 0.05) between the groups.

### 4.2. Flow Cytometry

Peripheral venous blood from all the qualified patients was collected into lithium heparin tubes and incubated at 37 °C in 5% CO_2_ in RPMI 1640 medium (Biowest, Nuaillé, France) (supplemented with 10% FBS (Biowest, Nuaillé, France), L-glutamine (Biowest, diluted 1:100, France), penicillin/streptomycin stock (Biowest, Nuaillé, France)) with 1 μL Leukocyte Activation Cocktail (LAC; 0.1 μg/mL phorbol 12-myristate 13-acetate (PMA, Cayman, Ann Arbor, MI, USA) + 1 μg/mL ionomycin (Cayman, USA)), 10 μg/mL Brefeldin A (BioLegend, San Diego, CA, USA), and 2 μM Monensin A (BioLegend, San Diego, CA, USA) for 4 h. No unstimulated control samples were included in this study. After stimulation, samples were stained with Zombie Red^TM^ Fixable Viability Kit (Biolegend, San Diego, CA, USA) for 15 min at 4 °C in the dark and then blocked with Human TruStain FcX™ (Fc Receptor Blocking Solution, Biolegend, San Diego, CA, USA) for 10 min at 4 °C in the dark. Surface staining was performed for 30 min at 4 °C with an appropriate cocktail of fluorescently labeled antibodies for regulatory T and B cells—anti-human CD19 (BioLegend, San Diego, CA, USA, clone: HIB19), CD24 (BioLegend, San Diego, CA, USA, clone: ML5), CD27 (BioLegend, San Diego, CA, USA, clone: M-T271), CD38 (BioLegend, San Diego, CA, USA, clone: HB-7) PD-L1 (BioLegend, San Diego, CA, USA, clone: 29E.2A3), PD-L2 (Becton Dickinson, Franklin Lakes, NJ, USA, clone: MIH18), CD45 (BioLegend, San Diego, CA, USA, clone: HI30), CD3 (BioLegend, San Diego, CA, USA, clone: UCHT1), CD4 (BioLegend, San Diego, CA, USA, clone: A161A1), CD127 (BioLegend, San Diego, CA, USA, clone: A019D5), PD-1 (BioLegend, San Diego, CA, USA, clone: EH12.2H7) and appropriate isotype controls (see Supplementary Table S1). The cells were then washed twice, and intracellular staining was performed according to the manufacturer’s protocol (True-Nuclear Transcription Factor Buffer Set, BioLegend, San Diego, CA, USA). Briefly, the cells were fixed for 14 h at 4 °C in the dark, washed twice with permeabilization buffer, blocked with Human TruStain FcX™ (BioLegend, San Diego, CA, USA) for 10 min at 4 °C in the dark and then stained for 1 h at 4 °C in the dark with anti-human Ebi3 (BioLegend, San Diego, CA, USA, clone: B032F6), p35 (R&D Systems, Minneapolis, MN, USA, clone: ABAR0216121), IL-10 (BioLegend, San Diego, CA, USA, clone: JES3-9D7), TGF-β (BioLegend, San Diego, CA, USA, clone: TW4-2F8) and Foxp3 (BioLegend, San Diego, CA, USA, clone: 206D) antibodies or appropriate isotype controls at the same concentration as the specific antibodies. IL-35-producing cells were defined based on intracellular co-expression of its two subunits, Ebi3 and p35. A complete list of antibodies and isotope controls used in the study is provided in Table S1 in the Supplementary Data. After washing twice with permeabilization buffer, the samples were analyzed using an LSR Fortessa Cell Analyzer (Becton Dickinson, Franklin Lakes, NJ, USA). A total of 150,000 viable events were recorded at a rate of 600–800 events per second. Data were analyzed using FlowJo software version 10.8.1 (Becton Dickinson, Franklin Lakes, NJ, USA). The frequencies of Breg subsets, including B10-like cells (CD19^+^CD24^high^CD27^+^), immature B cells (CD19^+^CD24^high^CD38^high^), and plasmablasts (CD19^−^CD27^int^CD38^+^), and the percentage of these cells expressing IL-35 (Ebi3^+^p35^+^), IL-10, PD-L1, and PD-L2 in women following miscarriage and pregnancy were examined. B10-like cells producing IL-10 were designed as B10 cells. The proportion of Tregs (CD45^+^CD3^+^CD4^+^CD127^−^Foxp3^+^) expressing IL-35, IL-10, TGF-β, and PD-1, as well as iTr35, Tr1, and Th3 cells expressing the aforementioned cytokines, were also analyzed.

### 4.3. Statistical Analysis

All statistical analyses were performed using GraphPad Prism 5.0.3. The shape of the data distribution was assessed using the Shapiro–Wilk or Kolmogorov–Smirnov normality test, and homoscedasticity was examined using Bartlett’s test. Normally distributed data from the two tested groups (normal pregnancy vs. pregnancy loss) were examined using Student’s *t*-test (parametric), and non-normally distributed data were analyzed using the Mann–Whitney U test (non-parametric). We used the Kruskal–Wallis test (nonparametric) with Dunn’s multiple comparison post-hoc test or one-way analysis of variance (parametric) with Tukey’s multiple comparison test to compare data from the three tested groups (normal pregnancy (NP) vs. one pregnancy loss (PL1) vs. 2–4 pregnancy losses (RPL)). The diagnostic significance of the ratio of the percentage of iTr35 cells to the percentage of IL-35^+^ cells was evaluated separately for immature cells, B10-like cells, and plasmablasts by receiver operating characteristic (ROC) curve analysis. ROC curves were generated for the comparisons NP vs. PL1 and NP vs. RPL. The optimal cut-off value was determined using the maximum Youden index, calculated as J = sensitivity + specificity − 1, providing the best balance between sensitivity and specificity. The distribution of women in individual subgroups by age, body mass index (BMI), gestational age, and number of pregnancies was tested using the chi-square test. Spearman’s correlation analysis was performed using RStudio 2023.12.1+402 software to assess correlations between Breg and Treg frequencies. *p* < 0.05 was considered statistically significant. The differences in correlation strength between groups and correlation coefficients were compared using Fisher’s z-transformation.

## Figures and Tables

**Table 1:** Spearman rank correlations and comparison of correlation coefficients between the NP group and the PL and RPL groups.

Variable Pair	rho NP	*p*-Value NP	rho PL	*p*-Value PL	rho RPL	*p*-Value RPL	Fisher’s Z NP vs. PL	Fisher’s Z NP vs. RPL	*p*-Value NP vs. PL	*p*-Value NP vs. RPL
% IL-35^+^ Immature cells vs. % iTr35 cells	0.27	0.03	0	0.972	0.27	0.248	1.639	0.000	0.101	1.000
% IL-35^+^ B10-like cells vs. % iTr35	0.28	0.021	0.14	0.213	−0.38	0.106	0.869	2.740	0.385	0.006
% IL-35^+^ plasmablasts vs % iTr35	0.41	0.001	0	0.997	−0.09	0.715	2.578	2.095	0.010	0.036
% PD-L1^+^ B10-like cells vs. % Treg	−0.34	0.005	0.09	0.447	−0.02	0.935	−2.630	−1.331	0.009	0.183
% PD-L1^+^ B10-like cells vs. % IL-35^+^ Treg	−0.34	0.004	0.1	0.406	−0.03	0.904	−2.690	−1.291	0.007	0.197
% PD-L1^+^ B10-like cells vs. % IL-10^+^ Treg	−0.3	0.012	0.11	0.329	0.11	0.623	−2.486	−1.673	0.013	0.094
% PD-L1^+^ B10-like cells vs. % TGFβ^+^ Treg	−0.36	0.003	0.14	0.242	0.1	0.652	−3.065	−1.901	0.002	0.057
% PD-L1^+^ B10-like cells vs. % Th3	−0.37	0.002	−0.12	0.326	0.09	0.718	−1.585	−1.907	0.113	0.057

rho—ρ, Spearman’s rank correlation coefficient; Z, test statistic based on Fisher’s z-transformation; *p*, statistical significance of the between-group difference in correlation coefficients. Between-group comparisons were performed for NP vs. PL and NP vs. RPL.

**Table 2:** Summary of ROC curve analysis. Data presents the area under the ROC curve (AUC), 95% confidence interval (CI), optimal cut-off value, sensitivity, specificity, and corresponding *p*-value for each analyzed cell population.

Parameter	AUC	AUC with 95% Confidence Interval	Cut-Off Point	Sensitivity	Specificity	*p*-Value
NP vs. PL1						
% iTr35/% IL-35^+^ immature cells	0.7328	0.6387 to 0.8269	0.74	0.4902	0.8889	<0.0001
% iTr35 cells/% IL-35^+^ B10-like cells	0.7405	0.6505 to 0.8306	0.45	0.4808	0.8657	<0.0001
% iTr35 cells/% IL-35^+^ plasmablasts	0.6483	0.5513 to 0.7453	10.2	0.4627	0.8182	0.0049
NP vs. RPL						
% iTr35/% IL-35^+^ immature cells	0.8532	0.7670 to 0.9393	1.74	1	0.57	<0.0001
% iTr35 cells/% IL-35^+^ B10-like cells	0.6835	0.5307 to 0.8363	0.9	0.6471	0.6418	0.0200
% iTr35 cells/% IL-35^+^ plasmablasts	0.6172	0.4850 to 0.7493	9.28	0.75	0.4925	0.1132

**Table 3:** The characteristics of the patients qualified for the study. *N*—group size, *n*—number of patients in the subgroup. Data are expressed as mean ± SD. chi^2^ test: *p* > 0.05 for patients’ age, BMI, pregnancy week and number of pregnancies.

	Normal Pregnancy *N* = 68 (% (*n*/*N*))	Pregnancy Loss *N* = 79 (% (*n*/*N*))
**Age (mean ± SD)**	**29.1 ± 5.4**	**31.6 ± 4.2**
18–29	44.0 (29/66)	32.0 (25/78)
30–36	47.0 (31/66)	59.0 (46/78)
37–41	9.0 (6/66)	9.0 (7/78)
**BMI, kg/m^2^ (mean ± SD)**	**23.1 ± 3.1**	**22.4 ± 2.7**
Underweight (Moderate thinness) (16.0–16.99)	2.9 (2/68)	1.2 (1/79)
Underweight (Mild thinness) (17.0–18.49)	2.9 (2/68)	5.0 (4/79)
Normal range (18.5–24.99)	69.1 (47/68)	77.2 (61/79)
Overweight (Pre-obese) (25.0–29.99)	25 (17/68)	16.5 (13/79)
**Pregnancy weeks (mean ± SD)**	**9.4 ± 2.1**	**9.3 ± 1.8**
5–8	38.2 (26/68)	31.6 (25/79)
9–13	61.8 (42/68)	68.4 (54/79)
**Miscarriages in the past**	**0.0 ± 0.0**	**1.4 ± 0.6**
1	**-**	70.89 (56/79)
2–4 (RPL)	**-**	29.11 (23/79)
**Number of pregnancies**	**1.8 ± 1.1**	**1.9 ± 1.0**
1	48.5 (33/68)	40.5 (32/79)
2	30.8 (21/68)	32.9 (26/79)
>2	20.6 (14/68)	26.6 (21/79)

## Data Availability

The data presented in this study are available on request from the corresponding author.

## Supplementary Materials

The following supporting information can be downloaded at https://www.mdpi.com/article/10.3390/ijms27209207/s1.
