# Supplementary material for: IL-35-Producing Regulatory B Cells Are Positively Correlated with iTr35 Cells in Healthy Pregnancy with Altered Associations Following Pregnancy Loss
Source: Int J Mol Sci. 2026 Oct 16;27(20):9207. doi: 10.3390/ijms27209207 (PMC13705534; doi:10.3390/ijms27209207)
Supplement: Supplementary file 1 [file ijms-27-09207-s001.zip › Supplementary data/Table S1.pdf]

Target	Fluorochrome	Isotype	Clone	Concentration (μg/test)	Catalog No.	Supplier
CD19	Alexa Fluor 488	Mouse IgG1, κ	HIB19	0,2	302219	BioLegend
Mouse IgG1, κ	Alexa Fluor 488		MOPC-21	0,2	400134	BioLegend
CD24	APC-Cy7	Mouse IgG2a, κ	ML5	0,3	311132	BioLegend
Mouse IgG2a, κ	APC-Cy7		MOPC-173	0,3	400230	BioLegend
CD27	PE-Cy7	Mouse IgG1, κ	M-T271	0,1	356412	BioLegend
Mouse IgG1, κ	PE-Cy7		MOPC-21	0,1	400126	BioLegend
CD38	Alexa Fluor 700	Mouse IgG1, κ	HB-7	0,05	356624	BioLegend
Mouse IgG1, κ	Alexa Fluor 700		MOPC-21	0,05	400180	BioLegend
PD-L1 (CD274)	Brilliant Violet 650	Mouse IgG2b, κ	29E.2A3	0,5	329740	BioLegend
Mouse IgG2b, κ	Brilliant Violet 650		MPC-11	0,5	400352	BioLegend
PD-L2 (CD273)	Brilliant Violet 510	Mouse IgG1, κ	MIH18	0,6	742717	Becton Dickinson
Mouse IgG1, κ	Brilliant Violet 510		X40	0,6	562946	Becton Dickinson
IL-10	Brilliant Violet 421	Rat IgG1, κ	JES3-9D7	0,2	501422	BioLegend
Rat IgG1, κ	Brilliant Violet 421		RTK2071	0,2	400439	BioLegend
Ebi3	PE	Mouse IgG2b, κ	B032F6	0,5	360904	BioLegend
Mouse IgG2b, κ	PE		MPC-11	0,5	400314	BioLegend
P35	APC	Mouse IgG1	-	10 µl/test	IC2191A	R&D
Mouse IgG1	APC		-	10 µl/test	IC002A	R&D
CD45	Brilliant Violet 785	Mouse IgG1, κ	HI30	0,05	304048	BioLegend
Mouse IgG1, κ	Brilliant Violet 785		MOPC-21	0,05	400170	BioLegend
CD3	Brilliant Violet 510	Mouse IgG1, κ	UCHT1	0,1	300448	BioLegend
Mouse IgG1, κ	Brilliant Violet 510		MOPC-21	0,1	400172	BioLegend
LAP/TGFβ1	PE/Cy7	Mouse IgG1, κ	FNLAP	0,25	25-9829-42	Invitrogen
Mouse IgG1, κ	PE/Cy7		P3.6.2.8.1	0,25	25-4714-80	Invitrogen
CD4	Alexa Fluor 700	Rat IgG2b, κ	A161A1	0,15	357418	BioLegend
Rat IgG2b, κ	Alexa Fluor 700		RTK4530	0,15	400628	BioLegend
CD127	PerCP/Cy5.5	Mouse IgG1, κ	A019D5	0,2	351322	BioLegend
Mouse IgG1, κ	PerCP/Cy5.5		MOPC-21	0,2	400150	BioLegend
FoxP3	Alexa Fluor 488	Mouse IgG1, κ	206D	0,5	320112	BioLegend
Mouse IgG1 κ	Alexa Fluor 488		MOPC-21	0,5	400134	BioLegend
PD-1	Brilliant Violet 650	Mouse IgG1, κ	EH12.2H7	0,3	329950	BioLegend
Mouse IgG1, κ	Brilliant Violet 650		MOPC-21	0,3	400164	BioLegend

Table S1. The complete list of antibodies and isotope controls used in the study.
